# Molecular Detection of *Malpighamoeba mellificae* in Honey Bees

**DOI:** 10.3390/vetsci9030148

**Published:** 2022-03-21

**Authors:** Marc O. Schäfer, Juliane Horenk, Claudia Wylezich

**Affiliations:** 1 Institute of Infectology, Friedrich-Loeffler-Institut (FLI), 17493 Greifswald, Germany; juliane.horenk@fli.de; 2 Institute of Diagnostic Virology, Friedrich-Loeffler-Institut (FLI), 17493 Greifswald, Germany; claudia.wylezich@fli.de

**Keywords:** amoebiasis disease, Malpighian tubules, adult honey bee, diagnostics, RT-qPCR, MAD-18S assay

## Abstract

*Malpighamoeba mellificae* is a protozoan that infects the Malpighian tubules of honey bees. The amoebae, ingested as cysts, develop into trophozoites that feed upon tubule epithelia. The resulting damage of the Malpighian tubules can induce an imbalance of waste excretion and hemolymph exchange. This causes the so-called amoebiasis disease in adult bees, which may co-occur with *Nosema* infections. Most reports of this amoeba are from the 1960s and earlier, and knowledge of the disease and its spreading is very poor. The lack of any genetic marker for the species hampers its sensitive identification using molecular tools and gaining knowledge on its epidemiology. Here, we present a diagnostic RT-qPCR assay, consisting of two primers and one probe that were developed based on 18S rRNA sequences of the amoeba, generated with metagenomic sequencing of Malpighian tubules with and without *M. mellificae* cysts. The assay was initially tested and adjusted with samples microscopically tested for the presence of *M. mellificae* cysts. Later, it was validated and material with unknown infection status was tested. The sensitive diagnostic *Malpighamoeba* disease 18S assay is now ready to be applied for honey bee health monitoring purposes and to investigate the prevalence of *M. mellificae* in more detail.

## 1. Introduction

Analogous to vertebrate kidneys, the principal organs of excretion and osmoregulation in insects are the Malpighian tubules [1,2]. Besides their classical role in osmoregulation, they have a high degree of specialization for organic solute transport, and for metabolism and detoxification [3]. These slender tubules have no muscle but move passively around the abdomen between other more mobile structures. In honey bees (*Apis mellifera*) they join at the junction from the midgut to hindgut, lying within the haemolymph, from which they filter excretory waste products. These excretory substances are then passed through the cellular lining of the tubules to the tubular canal and down the tubules to the gut, where the substances join undigested food as it enters the hindgut [4,5].

Infection with the entomopathogenic amoeba *Malpighamoeba mellificae* Prell, 1926, first described by Maaßen in 1916, can cause damage to these tubules [6,7,8]. The amoebae, once ingested by adult honey bees as cysts, develop into trophozoites that feed upon tubule epithelia [8]. The resulting damage of the Malpighian tubules can induce an imbalance of waste excretion and hemolymph exchange, which causes the so-called amoeba or amoebiasis disease of adult honey bees that was often shown to co-occur with *Nosema apis* infections [6,9]. Thus, it was shown in England (1954–1958), where apparently healthy honey bee colonies (*n* = 1759) were inspected for infection with *N. apis* and *M. mellificae*, that 43.9% of the colonies were infected with *N. apis* alone, 6.8% with *M. mellificae* alone, and 5.3% with both parasites [9]. When in Italy (1953–1957), individual bees (*n* = 52.484) from apparently healthy colonies were inspected for infection with *M. mellificae*, 1.1% were found positive and of these, 82.4% were infected with *M. mellificae* only, 10.4% were infected with both *M. mellificae* and *N. apis*, and 7.2% were positive for *M. mellificae* and *Acarapis woodi* [10]. In Italy, also a loss in honey productivity of up to 80% less than expected was observed in apiaries affected by *M. mellificae* [10]. Infections with *M. mellificae* have been suggested to be associated with spring dwindling, dysentery, and to shorten the lifespan of infected bees [10]. However, evidence to support these suggestions is rare, as the laboratory diagnostics to detect infections with *M. mellificae* are labor intense and therefore not regularly performed. The usual microscopic examination is carried out on fresh unstained material and relies completely on the detection of *M. mellificae* cysts.

Amoebae in general usually go through prolonged periods in the trophozoite state and encyst only in response to unfavorable conditions such as poor nutrition or desiccation [11]. While the factors causing encystment are still unknown in *M. mellificae*, it is possible that the actual occurrence of the disease is found underdiagnosed, if the amoeba indeed exists for long periods without forming cysts. As in other entomopathogenic amoebae, the detection of the disease is almost exclusively based on microscopic observation, due to the defined and constant morphology of the cysts and its marked light-refraction abilities [12]. In the absence of the cysts, the amoebiasis disease, although it might be present, could go unnoticed given the difficulty to distinguish trophozoites from the other normal content of Malpighian tubules, especially if the intensity of the infection is low [13]. In order to generate more accurate diagnoses, the development of molecular techniques that allow a fast, sensitive, and selective detection of all life stages of the amoeba is strongly required [13].

The aim of this study was the sensitive identification of *M. mellificae* using molecular tools. We therefore developed a diagnostic real-time (TaqMan) PCR tool based on 18S rRNA sequences generated with meta-ribosomalomics [14] of Malpighian tubule samples infected with the amoeba and not infected samples. This unbiased approach allows exploiting RNA-based metagenomics datasets for ribosomal sequences. After metagenomic sequencing, 18S rRNA and other gene sequences of the amoeba were revealed for the first time. The taxonomic assignment of 18S rRNA and actin was possible while further analysis is ongoing (Wylezich et al., manuscript in preparation). PCR primers and a probe for the obtained 18S rRNA of *Malpighamoeba* were developed, initially tested, and adjusted with samples microscopically tested for the presence of *M. mellificae* cysts.

## 2. Materials and Methods

### 2.1. Tested Sample Material

Malpighian tubules and intestines of individual honey bees (*Apis mellifera*) were initially screened via a light microscope (Axio Lab, Carl Zeiss Microscopy GmbH, Jena, Germany) to ensure the presence of *Malpighamoeba* cysts. All samples were prepared in PBS. If the material was stored overnight at 4 °C, samples were centrifuged (5 min at 6.797× *g*) and the PBS supernatant was tested via PCR as well. Later, pools of abdomen homogenates were tested to investigate whether the preparation of Malpighian tubules can be circumvented. In addition, intestine pools (stored at −80 °C), which were proven to be infected with *Nosema* spp., were used for *Malpighamoeba* monitoring without prior microscopic screening for *M. mellificae* cysts. In one case in spring 2021, fecal spots were sampled from the bottom board insert of a honey bee colony that was suspected to be infected with *M. mellificae* and/or *Nosema* spp. due to fecal contamination inside the colony and around the hive entrance. Bees without microscopically detectable *M. mellificae* infection (absence of cysts) were used as a negative control (NC; Malpighian tubules or entire abdomen). In addition, head or thorax samples of honey bees with unknown *Malpighamoeba* infection status were tested as NC since the amoebae are typically associated with the Malpighian tubules inside the abdomen.

In addition to the *Malpighamoeba* sample material, stored DNA isolated from other amoeba (cultivated strains) were used to check the specificity of PCR primers, the probe, and the PCR conditions. These were *Echinamoeba* sp., *Flamella aegyptia*, *Saccamoeba stagnicola*, *Sappinia* sp., *Vannella miroides*, *Vannella* sp. and *Thecamoeba striata*.

### 2.2. Sample Disintegration and Nucleic Acid Extraction

Prepared Malpighian tubules of bees were homogenized with 200 µL 1× PBS and four steel grinding balls (2 mm diameter) in 0.5 mL tubes using a vortex mixer at full speed for 1 min. Single intestine samples were treated similar to Malpighian tubules but with 300 µL 1× PBS and two 3 mm grinding balls. Single abdomen samples were disintegrated in 2 mL tubes with the aid of a TissueLyser II (Qiagen, Hilden, Germany) with 500 µL 1× PBS and steel grinding balls (2 × 3 mm and 1 × 5 mm) at 30 Hz for 2 min. Intestine pools were treated similar to abdomen samples but with 1 mL 1× PBS and four steel balls (3 mm diameter). Abdomen pools or entire bees were disintegrated in an M Tube with 500 µL 1× PBS or ZB5d medium (=minimum essential medium with Earle’s and Hank’s salts) per sample using gentleMACS (Miltenyi Biotech, Bergisch-Gladbach, Germany) applying the program RNA 02.01, M Tube. From the fecal spots, some material was mixed with 400 µL 1× PBS and vortexed for about 3 min until it was dissolved completely. All samples except abdomen pools or entire bees were afterwards centrifuged at 6.797× *g* for 3 min to pellet debris. Abdomen pools or entire bees were centrifuged at 266× *g* for 10 min. One hundred microliters of the homogenate (Malpighian tubules, abdomen, intestine, and intestine pools) or 150 µL (entire bees, abdomen pools, and fecal spots) was introduced to RNA extraction. RNA of all described sample material was extracted using the RNeasy Mini Kit (Qiagen) according to the manufacturer’s instructions with 60 µL (Malpighian tubules, intestine and intestine pools, fecal spots) or 100 µL (abdomen, abdomen pools, or entire bees) final RNA eluate. Internal control RNA, covering a 712 bp fragment of the enhanced green fluorescent protein (EGFP) gene, was added to all samples before extraction [15]. Furthermore, negative RNA isolation controls (=RIC; pig serum instead of honey bee homogenate) were taken along in adequate number (integrated after every 7th sample), during each extraction process to preclude contamination-based PCR products.

### 2.3. RT-qPCR Assay for Malpighamoeba mellificae

For the RT-qPCR assay to monitor the amoebiasis disease in adult bees, different PCR primers and an oligonucleotide probe were developed based on the 18S rRNA gene of *Malpighamoeba mellificae* (GenBank accession number OL757386). The final oligonucleotides used are given in Table 1. Primer sequences for the internal control RNA (EGFP-Mix 1 = EGFP-1-F: 5′-GAC-CAC-TAC-CAG-CAG-AAC-AC-3′; EGFP-2-R: 5′-GAA-CTC-CAG-CAG-GAC-CAT-G-3′ and 5′-HEX-AGC-ACC-CAG-TCC-GCC-CTG-AGC-A-BHQ1-3′) were taken from Hoffmann et al. [15].

**Figure 1:**
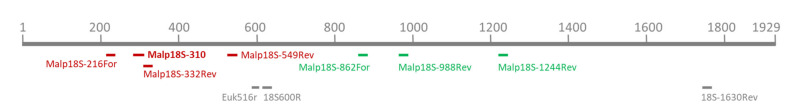
Depiction of primer binding sites of all primers and the probe used in this study. The upper scale represents the 18S rRNA gene of *M. mellificae*, length given in base pairs. *Malpighamoeba* specific primers used as mix A1 and A2 (red-marked with the probe, marked in bold letters) or mix C1 and C2 (green-marked; compare Table 1 for sequences) and eukaryote-specific primers (grey-marked) are given.

Oligonucleotides were checked via blast search to avoid cross-specificity with the host and other taxa that might be connected with bees (e.g., *Nosema* spp.). Several regions of the gene were used for primer design (Figure 1). The AgPath-ID One-Step RT-PCR (ThermoFisher Scientific, Dreieich, Germany) according to the manufacturer’s instructions and a BioRad CFX96 Real-Time Detection System (Bio-Rad, Hercules, CA, USA) were applied. In brief, 2.5 µL extracted RNA or DNA was combined with 6.25 µL 2× RT-PCR Buffer, 0.5 µL 25× RT-PCR Enzyme Mix, 0.5 µL of the forward and reverse primer (10 µM), 1 µL EGFP-Mix 1 [15], and filled up with nuclease-free water to a final volume of 12.5 µL per sample. Cycling conditions were the following: reverse transcription at 45 °C 10 min, 10 min at 95 °C, followed by 35 cycles consisting of 15 s at 94 °C, 20 s at 61.8 °C, 15 s at 72 °C. A no-template control (NTC) and a background control were included in the PCR runs. The background control was made from microscopically *Malpighamoeba* cyst-negative material additionally checked negative via PCR. Gradient PCR was applied to optimize the annealing temperature using a C1000 Real-Time Detection System (Bio-Rad, Hercules, CA, USA). To adjust the PCR conditions and find suited primer pairs detecting *Malpighamoeba* and avoiding cross-specificity with the host or other taxa in the sample, the above-described PCR was modified as follows: 0.625 µL LightCyler 480 ResoLight Dye (Roche Diagnostics) were added to each PCR reaction and the cycling conditions were supplemented by a melting curve (1 s at 65–95 °C with an increment of 0.2 °C). PCR products were checked on a 1% agarose gel and selected bands were excised. For the final RT-qPCR, mix A primers (Table 1; A1 and A2) in combination with the probe Malp18S-310R-FAM (0.25 µL per PCR reaction, 10 µM) and the EGFP-Mix 1 supplemented by the EGFP-HEX probe [15] were applied with the abovementioned reagents and conditions but with 40 amplification cycles instead of 35. Fluorescence was recorded during the annealing step. Specificity was tested with stored DNA of different amoeba strains and with obviously not infected bees or parts of bees (thorax, head). For this purpose, primer pairs A1 and A2 with the probe (Table 1) were applied. A dilution series of a purified PCR product (generated of *Malpighamoeba* cyst-positive material using the primer pair A1 and subsequent purification of the PCR product using AMPure XP beads, Beckman Coulter) was used to test the analytical sensitivity and limit of detection (LOD). For this purpose, the PCR product was diluted in background control.

To verify the PCR products, excised amplicons were purified using the QIAquick Gel Extraction Kit (Qiagen, Hilden, Germany). Eluates were sent to Eurofins for Sanger sequencing. Obtained sequences were trimmed, corrected, and submitted to NCBI (accession numbers OL757386-OL757399).

### 2.4. Sequence Analysis of Amplified Partial Malpighamoeba 18S rRNA Sequences

18S rRNA fragments of *M. mellificae* generated with the primers of mix A and mix C (Table 1; A1, A2, C1, and C2) were visualized and compared with each other and with the complete 18S rRNA sequence of *M. mellificae* (accession number OL757386) using Geneious version 10.2.3 (Biomatters, Auckland, New Zealand). Sequences were aligned together using MAFFT version 7.388 [19] as implemented in Geneious.

## 3. Results

### 3.1. Sequence Diversity of Malpighamoeba mellificae

The comparison of the metagenomics-derived 18S rRNA sequence (Wylezich et al., manuscript in preparation) with the Sanger-sequenced fragments generated with primer mix A2 showed identical sequences. Consensus sequences generated with mix C2 showed two variations, namely two transitions at position 1068 (A→G) and position 1215 (C→T). Both variations are ambiguities and were depicted in the sequences as R and Y, respectively. The primer and probe binding sites are not affected by these single nucleotide variants.

### 3.2. Diagnostic RT-qPCR for Malpighamoeba

The duplex RT-qPCR assay for detection of the *Malpighamoeba mellificae* disease (MAD-18S assay) is the one we presented in paragraph 2.3. with primer pair A1 (generating a 117 base pair PCR product) and probe Malp18S-310R-FAM with 40 amplification cycles in combination with the EGFP-1 primer mix generating a PCR product of comparable length (132 bp). The application as a singleplex assay (without the EGFP-1 primer mix) is possible and would not influence the sensitivity of the detection of *M. mellificae*. Moreover, primer pair A2 with the probe would result in reliable measurements.

### 3.3. Specificity of MAD-18S Assay

The DNA of amoebae other than *Malpighamoeba* (cultured strains) were diluted at 1:100 and 1:1000. They were tested with primer mixes A1 and A2 including the probe and resulted in no quantification cycle (Cq) value and no PCR product for any sample as checked additionally on an agarose gel. There was also no cross-reactivity with host nucleic acids since non-infected bees (Malpighian tubules of a single bee and the background control) resulted in no Cq value and no PCR products. In addition, the specificity of the assay was also confirmed by sequencing of the generated PCR products of sample material with positive and unknown infection status. Head and thorax samples of bees resulted in no Cq value and no PCR products and might serve as negative controls.

### 3.4. Sensitivity of MAD-18S Assay

**Figure 2:**
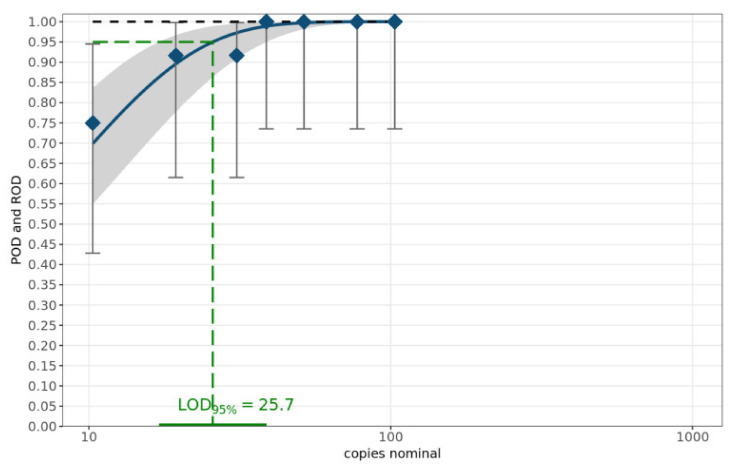
POD curve and LOD_95%_. The blue diamonds characterize the laboratory-specific rate of detection (ROD). The blue curve denotes the mean POD curve along with the corresponding 95% confidence range highlighted as the grey band. The POD curve under ideal conditions is displayed as the black dashed curve.

The theoretical number of copies of the target sequence was calculated according to the length of the sequence (in base pairs) and nucleic acid concentrations in sample volumes of one microliter, measured by spectrophotometric determination using NanoDrop (ThermoFisher, Darmstadt, Germany) [20,21]. The sensitivity of the MAD-18S assay was determined with a dilution series of 7 dilutions of the amplicon of sample c1-2020-Mt4 (Table 2) using primer mix A1 and performed on 12 replicates per dilution level [20]. The limit of detection (LOD_95%_) was determined using a web service provided by the German Federal Office of Consumer Protection and Food Safety (BVL) [21]. This web service for the validation of qualitative PCR methods within a single laboratory applies a statistical model for calculating the probability of detection (POD) curve and the number of copies of the target sequence required to ensure a 95% POD. Figure 2 summarizes the results. The LOD_95%_ is 25.7 copies of 18S rRNA per µL with a 95% confidence interval of (17.1, 38.8).

### 3.5. Investigation of Different Sample Matrices

The MAD-18S assay was tested with different bee sample material: prepared Malpighian tubules of single bees, PBS supernatant of these preparations, intestine of single bees (*n =* 1) and intestine pools of 20 bees (*n =* 20), abdomen pools of 20 bees (*n =* 20), and one feces sample. The microscopically verified, clearly infected samples showed positive Cq values between 8.5 and 19.8, except older dried-up samples that had to be soaked in PBS, which showed Cq values between 20.7 and 32.1 (c6-2019; Table 2). Microscopically screened samples that were negative for *M. mellificae* cysts showed a Cq value for EGFP, which was comparable with the positive sample material (see c7-2021-Mt in Table 2). Altogether, more than 200 samples with an unknown infection status were tested. Those that tested positive for *M. mellificae* showed Cq values between 18.1 (c11-2021-ap) and 26.0 (c12-2021-ip; Table 2).

## 4. Discussion

To date, there have been no molecular studies on *M. mellificae*, besides the own study (Wylezich et al., manuscript in preparation), and to the best of our knowledge, no published PCR detection assays currently exist. In this study, TaqMan RT-qPCR was used successfully to enable species identification and detection of *M. mellificae* in honey bees, without the need for labor-intensive microscopic observation. In our study, the primers and probe were designed to sequence a 117 bp-long PCR product of the 18S rRNA. Our MAD-18S assay for the detection of the *Malpighamoeba* disease showed positive results with different bee sample material including prepared Malpighian tubules, supernatant of these preparations, intestine of single bees, intestine pools, abdomen pools but not head or thorax samples. Molecular detection of *Malpighamoeba* was possible even with a sample of feces taken from the bottom board insert of a colony that showed symptoms of amoeba disease. The approach is applicable as a duplex or singleplex assay and works with DNA and RNA as starting material. The Cq values obtained for the different matrices (Table 2) recommend the applicability of the assay to complex samples (abdomen, intestine, feces) instead of only prepared Malpighian tubules, as well as the possibility of pooled samples. The assay was shown to be sensitive with a LOD_95%_ of 25.7 rRNA copies per µL (Figure 2). The primers were shown to be specific for *M. mellificae*, as no cross-reaction was observed with RNA or DNA of homogenized honey bees or parts of honey bees as well as with DNA of amoebae other than *Malpighamoeba*. The results suggest that the MAD-18S assay is reliable for screening honey bee colonies for the presence of the target amoebae. This assay and its applicability for complex and pooled samples strongly facilitates the future monitoring of the *Malpighamoeba* and allows for the first time the collection of systematic data on that *Malpighamoeba* disease.

Most reports of *M. mellificae* are from the past and therefore knowledge of the amoeba or amoebiasis disease in adult bees and its spreading is very poor. After the disease was discovered one hundred years ago by Maaßen in Germany [7], it was reported in Switzerland [22], the United States [23], the United Kingdom [24], Italy [10] and since then in many other countries worldwide [25]. However, the disease received only limited attention, which is probably due to the fact that infections of honey bees with *M. mellificae* seem to play a minor role only as the course of the disease is rarely serious and it often disappears spontaneously. However, as detection was always based on microscopical observations of the cysts, the disease might have been overlooked even when it was present [13].

The role of protozoan parasites in honey bee health and their distribution across the world is not well understood. The herein described diagnostic PCR tool for the detection of *M. mellificae* will allow studying the so-called amoeba or amoebiasis disease in adult bees in large-scale surveillance studies as well as in routine diagnostics allowing to bring more light and understanding into the role of this protozoan on the health of honey bee colonies worldwide. It is now also possible to study the role of *Malpighamoeba* for the often-observed co-infection with *Nosema* species in more detail.

There is scope to improve and refine the method further including the reduction in background noise and increasing the amount of honey bees or volume homogenate tested at any one time. In addition, the method needs further validation with more infested samples of different origins to consider the overall *Malpighamoeba* genetic diversity and to verify the reliability of the assay. Although several samples were tested via sequencing to take sequence diversity into account, this was only restricted to sample material from Germany until now.

## Figures and Tables

**Table 1:** Primers specific for the 18S rRNA gene used in this study. *Malpighamoeba*-specific primers were used in combination as mix A1 and A2 (Malp18S-216For + Malp18S-332Rev or Malp18S-549Rev, respectively) or mix C1 and C2 (Malp18S-862For + Malp18S-988Rev or Malp18S-1244 Rev, respectively). 18S-1630Rev, Euk516r, 18S600R are universal eukaryotic primers. The probe Malp18S-310R-FAM was complemented with mix A1 and A2.

Name	Sequence (5′–3′)	Reference
Malp18S-216For	TATACAGATTGTGTAAAAGCG	This study
Malp18S-332Rev	TTAGCCTCTATCTAACCTACC	This study
Malp18S-549Rev	AAAGCATATCTCGGCATAACCG	This study
Malp18S-862For	GGGATTAGATGTATTGGTTGGC	This study
Malp18S-988Rev	AATCATCTTCGATCCTTATCCC	This study
Malp18S-1244Rev	TCCTACCTTGGTAAAATTTCCC	This study
Malp18S-310-FAM	Fam-TACAAGAGGATCTGCCCTATCAACTAT-Tamra	This study
18S-1630Rev	CGACGGGCGGTGTGTACAA	[16]
Euk516r	ACCAGACTTGCCCTCC	[17]
18S600R	GCTATTGGAGCTGGAATTACCG	[18]

**Table 2:** Results of the duplex MAD-18S assay for different sample material. Mean Cq values and standard deviations (in brackets) are given for 3 replicates each for the target *Malpighamoeba* and the EGFP internal control. The infection status “Verified” refers to a positive microscopical investigation prior to PCR. “Not verified” means that no microscopical investigation was implemented on this sample set of bees deriving from a positive colony. “Unknown” means that there was no information on the colony’s infection status. Abbreviations: Cq, quantification cycle; EGFP, enhanced green fluorescent protein.

Sample Name	Sample Material	Infection Status	Cq *Malpighamoeba*	Cq EGFP
c1-2020-Mt1	Malpighian tubules (*n* = 1)	Verified	8.5 (0.50)	27.4 (0.46)
c1-2020-Mt2	Malpighian tubules (*n* = 1)	Verified	12.8 (0.11)	26.2 (0.10)
c1-2020-Mt3	Malpighian tubules (*n* = 1)	Verified	9.5 (0.21)	27.8 (0.10)
c1-2020-Mt4	Malpighian tubules (*n* = 1)	Verified	9.4 (0.55)	26.2 (0.24)
c1-2020-ap1	Abdomen pool (*n* = 20)	Not verified	10.8 (0.33)	29.2 (0.18)
c1-2020-ap2	Abdomen pool (*n* = 20)	Not verified	12.3 (0.42)	27.1 (0.24)
c1-2020-ap3	Abdomen pool (*n* = 20)	Not verified	11.1 (0.30)	27.7 (0.12)
c1-2020-ap4	Abdomen pool (*n* = 20)	Not verified	8.9 (0.07)	27.6 (0.52)
c2-2019-Mt	Malpighian tubules (*n* = 1)	Verified	18.6 (0.20)	25.1 (0.06)
c2-2019-ap	Abdomen pool (*n* = 20)	Not verified	25.1 (0.82)	26.6 (0.22)
c3-2021-Mt	Malpighian tubules (*n* = 1)	Verified	16.7 (0.13)	23.7 (0.20)
c3-2021-ap	Abdomen pool (*n* = 20)	Not verified	17.9 (0.14)	24.3 (0.05)
c4-2017-Mt	Malpighian tubules (*n* = 1)	Verified	14.1 (0.74)	25.9 (0.63)
c4-2017-sup	Supernatant of Mt	Verified	19.8 (0.03)	27.1 (0.12)
c5-2021-Mt	Malpighian tubules (*n* = 1)	Verified	11.9 (0.19)	24.1 (0.09)
c5-2021-ap	Abdomen pool (*n* = 20)	Not verified	18.1 (0.26)	24.6 (0.14)
c6-2019-i1	Intestine (*n* = 1)	Verified	24.1 (0.24)	26.2 (0.12)
c6-2019-Mt1	Malpighian tubules (*n* = 1)	Verified	24.6 (0.32)	26.0 (0.24)
c6-2019-i2	Intestine (*n* = 1)	Verified	20.7 (0.30)	25.0 (0.06)
c6-2019-Mt2	Malpighian tubules (*n* = 1)	Verified	24.4 (0.39)	25.8 (0.05)
c6-2019-Mt3	Malpighian tubules (*n* = 1)	Verified	32.1 (0.80)	26.1 (0.37)
c6-2019-sup3	Supernatant of Mt	Verified	28.6 (0.36)	25.9 (0.04)
c7-2021-Mt	Malpighian tubules (*n* = 1)	Not infected	No Cq	24.1 (0.15)
c8-2017-Mt	Malpighian tubules (*n* = 1)	Verified	12.7 (0.25)	22.8 (0.14)
c8-2017-sup	Supernatant of Mt	Verified	17.2 (0.39)	25.5 (0.14)
c9-2020-ap	Abdomen pool (*n* = 20)	Unknown	23.1 (0.56)	29.1 (0.34)
c10-2020-ap	Abdomen pool (*n* = 20)	Unknown	19.1 (0.53)	28.8 (0.16)
c11-2021-ap	Abdomen pool (*n* = 20)	Unknown	18.1 (0.23)	24.1 (0.15)
c12- 2021-ip	Intestine pool (*n* = 20)	Unknown	26.0 (0.28)	22.0 (0.07)
c13-2021-f	Feces (fecal spots)	Unknown	25.2 (0.80)	22.0 (0.12)

## Data Availability

The data presented in this study are available in the article.
